# Prognostic Stratification of Multiple-Classifier Endometrial Cancers: Cohort Study and Meta-Analysis

**DOI:** 10.3390/cancers18060929

**Published:** 2026-03-12

**Authors:** Sabrina Paratore, Angela Russo, Katia Lanzafame, Giusi Blanco, Eliana Giurato, Giovanni Bartoloni, Marco D’Asta, Mirella Sapienza, Giulia Maria Bonanno, Antonino Vallone, Giuseppe Ettore, Roberto Bordonaro

**Affiliations:** 1 Pathological Anatomy Unit, Department of Oncology, ARNAS Garibaldi Hospital, 95122 Catania, Italy; a.russo@ao-garibaldi.ct.it (A.R.); gbartoloni@arnasgaribaldi.it (G.B.); 2 Medical Oncology Unit, Department of Oncology, ARNAS Garibaldi Hospital, 95122 Catania, Italy; klanzafame@arnasgaribaldi.it (K.L.); gblanco@arnasgaribaldi.it (G.B.); rbordonaro@arnasgaribaldi.it (R.B.); 3 Obstetrics and Gynecology Unit, Maternal-Infantile Department, ARNAS Garibaldi Hospital, 95122 Catania, Italymsapienza@arnasgaribaldi.it (M.S.); giuliabonanno@arnasgaribaldi.it (G.M.B.); gettore@arnasgaribaldi.it (G.E.); 4 Radiology Unit, ARNAS Garibaldi Hospital, 95122 Catania, Italy; avallone@arnasgaribaldi.it

**Keywords:** endometrial cancer, molecular profile, clinicopathological features, multiple-classifier, POLE mutated, mismatch repair-deficient, TP53mutated, next generation sequencing

## Abstract

**Background/Objectives**: The integration of molecular classification has significantly refined prognostic stratification in endometrial carcinoma (EC). However, a tumor subset harboring more than one molecular classifier challenges the current hierarchical classification systems, and their biological and clinical significance remains incompletely defined. **Methods**: We analyzed an expanded cohort of 150 EC patients using next-generation sequencing and immunohistochemistry, integrating molecular and clinicopathological data. Multiple-classifier ECs were identified and compared with those of single-classifier tumors sharing at least one molecular feature. A systematic review and meta-analysis including our cohort were also performed to estimate the incidence and distribution of multiple-classifier ECs. **Results**: In our cohort, 6% of ECs harbored molecular multiple-classifiers. POLEmut-MMRd/MSI tumors generally retained POLE-mutated features, including ultrahigh tumor mutational burden (TMB), early-stage disease, and favorable clinicopathological characteristics, consistent with a highly immunogenic phenotype. In contrast, MMRd/MSI-p53abn/TP53mut tumors were more frequently associated with adverse clinicopathological features and showed lower and heterogeneous TMB values, suggesting that the coexistence of TP53 alterations may modify the typical intermediate-risk of MMRd/MSI-only tumors. Triple-classifier tumors were exceedingly rare, precluding definitive conclusions regarding prognosis. The meta-analysis demonstrated an overall prevalence of multiple-classifier ECs of approximately 5.4%, with substantial inter-study heterogeneity largely attributable to differences in molecular testing strategies, analytical sensitivity, and variant interpretation criteria. **Conclusions**: Multiple-classifier ECs represent a small but clinically relevant subset encompassing biologically heterogeneous entities. Our findings highlight the limitations of current molecular classification hierarchies and underscore the need for harmonized molecular testing and standardized reporting to improve risk stratification and the management of multiple-classifier ECs.

## Simple Summary

The classification of endometrial cancer has evolved using molecular features, allowing for improved risk assessment and more personalized treatment strategies. However, a small proportion of tumors show more than one relevant molecular alteration, making their classification and clinical management more challenging. This study aimed to characterize molecular and clinicopathological profiles of multiple-classifier endometrial cancers, enhancing our understanding of their biological heterogeneity. In our patient cohort, POLEmut tumors with concurrent MMRd/MSI generally retained the POLE-associated ultramutated profile, while tumors with both MMRd/MSI and p53abn/TP53mut alterations were more often associated with more adverse clinicopathological characteristics compared to MMRd/MSI-only tumors. By integrating our data in a systematic literature review with meta-analysis, we observed that variability in reported incidence is largely driven by differences in testing strategies. These results highlight the limitations of current classification systems and emphasize the importance of more standardized molecular approaches to improve risk stratification and management in endometrial cancer.

## 1. Introduction

The molecular classification of endometrial carcinoma (EC), as established by The Cancer Genome Atlas (TCGA), represents a pivotal advance in gynecologic oncology [1]. By delineating four molecular subgroups—POLE-ultramutated, microsatellite instability-high (MSI-H), copy-number low (NSMP, no specific molecular profile), and copy-number high (p53-abnormal)—the TCGA taxonomy has provided a reproducible and prognostically robust framework compared to traditional histotype and grade-based models [1,2]. The integration of these classifiers into clinical guidelines (ESGO/ESTRO/ESP, NCCN) has improved risk stratification and guided the personalization of adjuvant therapy [3,4,5,6,7,8,9]. The TCGA-derived model assumes mutually exclusive molecular categories. However, the systemic application of molecular classification has highlighted the existence of a small but non-negligible fraction of tumors harboring more than one molecular alteration—the so-called “multiple-classifier” ECs [10,11,12]. Typical combinations include POLEmut-p53abn, MMRdp53abn, and POLEmut-MMRd, while triple-classifier tumors combining all three aberrations are rare [10]. Published studies estimate that multiple-classifier ECs account for approximately 3–11% of ECs [10,11,12]. The wide variability in the reported prevalence data reflects differences in testing depth, the addition of TP53 sequencing to immunohistochemistry (IHC), cohort composition (often enriched for high-grade tumors), and heterogeneous criteria for interpreting subclonal p53 staining [12,13,14,15]. Consequently, prevalence seems to depend not only on biological factors, but also the methodological approaches employed: centers using broader and more systematic molecular evaluation tend to identify a higher number of overlapping classifiers [12].

These hybrid cases raise critical questions, particularly regarding which classifier should drive adjuvant therapy. Current diagnostic algorithms, such as ProMisE (Proactive Molecular Risk Classifier for Endometrial Cancer), prioritize certain mutations to simplify clinical decision-making [9,16]. According to established clinical hierarchies, tumors harboring a pathogenic POLE exonuclease domain mutation are assigned to the POLE-ultramutated (POLEmut) group, regardless of the concomitant presence of MMRd or p53 abnormalities. In the absence of POLE mutations, tumors showing both MMRd and p53 abnormalities are classified as MMRd. However, emerging evidence indicates that multiple-classifier EC do not represent a single biological entity but rather encompass distinct genetic subgroups with heterogeneous clinicopathological behaviors [10,12,15]. In some combinations, one molecular signature appears to be biologically dominant and dictates clinical outcome—for example, POLEmut–p53abn tumors typically retain the favorable prognosis associated with POLEmut cancers. In other scenarios, the coexistence of molecular classifiers correlates with more aggressive disease, challenging the prognostic validity of the current hierarchical framework for these subgroups [10,12,17].

Given the relative rarity of multiple-classifier ECs and the still limited follow-up available in most studies, their prognostic stratification and optimal therapeutic management remain poorly defined. In this context, in the present study, we aimed to characterize molecular and clinicopathological profiles of multiple-classifier ECs, enhancing our understanding of their biological heterogeneity. Furthermore, to estimate their incidence, we performed a systematic literature review with meta-analysis, integrating data from previously published cohorts with those from our institutional series.

## 2. Materials and Methods

### 2.1. Case Selection

Our retrospective cohort consisted of 65 patients with ECs diagnosed at ARNAS Garibaldi Hospital from June 2024 to June 2025. According to the WHO Classification, 5th edition (2020), all hematoxylin and eosin slides were reviewed by two pathologists, and the diagnosis of EC was confirmed on the basis of morphologic features. For each case, clinical and molecular data were collected, including age, FIGO stage, histologic subtypes, Grade, Lymphatic Vascular Space Invasion (LVSI), lymph node status, and molecular subtypes. Informed consent to the data collection and analysis was obtained from all patients according to institutional guidelines. To expand cohort analysis and statistical evaluations, the data obtained from the 65 cases were integrated with a previously published database containing 85 EC cases with similar characteristics [18], resulting in a total of 150 cases.

### 2.2. Next Generation Sequencing and Immunohistochemistry

Molecular classification of EC patients (n = 65) was performed using a combined approach of Next Generation Sequencing (NGS) and immunohistochemistry (IHC). According to the criteria described in previous studies [10,19], the POLEmut patients were defined by mutations in the POLE gene exonuclease domain through NGS analysis. By NGS and IHC, the MSI subgroup was characterized by the presence of Microsatellite Instability (MSI) and/or by the identification of MMRd, the p53abn group showed aberrant expression patterns, and/or TP53 gene mutations.

For the NGS assay, six formaldehyde-fixed and paraffin embedded tissue (FFPE) sections of 5- to 10-μm-thin size, containing more than 50% tumor cells of EC patients were used for DNA extraction by the EZ1/EZ2 DNA FFPE Kit (Qiagen, Hilden, Germany), according to the manufacturer’s instructions. The initial amount of 10–50 ng of total DNA was required for library preparation with hybridization capture-based target enrichment using a commercially available 50-gene panel Myriapod NGS Cancer probe plus (Diatech Pharmacogenetics, Jesi, Italy) and following the manufacturer’s protocol. This panel covered hotspot mutation regions relevant to EC (including the TP53 and POLE exonuclease domains) and allowed for MSI analysis. Separate sequencing runs were performed by the MiSeq Dx platform (Illumina, Cambridge, UK) and analyzed by Myriapod NGS Data Analysis Software v 5.0.10. Variants were further filtered based on the following criteria: a minimum read depth of 200X; variant allele frequency (VAF) threshold of at least 5%; presence in both forward and reverse reads; exclusion of known sequencing artifacts and common polymorphisms (using databases such as gnomAD); annotation using curated databases (e.g., COSMIC, ClinVar) and bioinformatics pipeline (Varsome clinical v.12.9.0 software) was performed to assess pathogenicity. Data analysis focused on non-synonymous variants having functional consequences at the protein level (synonymous variants, uncertain significance variants (VUS), and intronic variants not affecting splice sites were excluded from the final analysis). Regarding MSI analysis, the MSI phenotype detection was based on the read count distribution of 120 specific microsatellite loci. A given threshold was set using the coverage ratio of a specific set of repeat lengths for each microsatellite locus. The locus was categorized as stable (MSS) if the coverage ratio was less than the given threshold. The MSI status was based on the percentage of unstable loci in the specific sample. A tumor sample was considered highly unstable (MSI) if >30% of the marker loci were length-unstable. For the previously published 85 cases, they had been classified according to the same WHO 2020 criteria and molecular stratification framework. For the purposes of the present study, raw sequencing data from the previously published cohort were retrieved and reprocessed using the identical bioinformatic pipeline adopted for the 65 newly analyzed cases. In particular, the same variant filtering thresholds, artifact exclusion criteria, polymorphism filtering, annotation databases, and pathogenicity classification strategy (Varsome Clinical software v.12.9.0) were uniformly applied to both datasets. Moreover, sequencing platform, gene panel design, MSI analytical algorithm, and quality control metrics (including coverage uniformity and mean depth) were comparable between the two cohorts, and no statistically significant differences in sequencing performance parameters were observed. This harmonization strategy ensured complete analytical consistency and minimized potential technical bias across the entire 150-case cohort.

A selected number of cases (n = 9), already included in the main cohort and previously analyzed using the same NGS workflow described above, underwent additional tumor molecular board (TMB) using the TruSight Oncology 500 multigene panel on the NexSeq NGS platform (Illumina, Cambridge), following the manufacturer’s protocol. Sequencing data analysis, including the identification of somatic mutations across 523 genes and TMB evaluation, was performed by Dragen (Dynamic Read Analysis for Genomics) software version v.2.6 (Illumina, Cambridge). To calculate the TMB, the total number of mutations was divided by the size of the panel (in millions of bases) and expressed as mutations per megabase (mut/Mb). A high TMB was defined as ≥10 mut/Mb.

For IHC analysis, 3-μm-thick FFPE sections of 65 EC samples were prepared. Primary antibodies used for the evaluation of mismatch repair (MMR) proteins were: MLH1 (clone: M1, Ventana Medical Systems, Tucson, AZ, USA), PMS2 (clone: A16-4, Ventana Medical Systems, Tucson, AZ, USA), MSH2 (clone: G219-1129, Ventana Medical Systems, Tucson, AZ, USA), and MSH6 (clone:SP93, Ventana Medical Systems, Tucson, AZ, USA), all ready-to-use on BenchMark IHC/ISH instruments with the OptiView DAB IHC Detection Kit, OptiView Amplification Kit, and ancillary reagents. The primary antibody for p53 staining was anti-p53 (DO-7, Ventana Medical Systems, Tucson, AZ, USA). Following previous published criteria [13], three p53 expression patterns were considered aberrant: strong diffuse nuclear staining in > 80% of tumor cells; complete absence of staining; and cytoplasmic staining. For both p53abn/TP53mut and MMRd/MSI classifications, cases were defined as positive if either IHC or NGS yielded a positive result. In the event of discordance between the two methods, NGS results were prioritized over IHC for tumor classification.

### 2.3. Literature Search Strategy

The primary literature search was performed in PubMed, focusing on English-language studies published within the five years prior to December 2025 reporting EC molecular classification. A full description of the search strategy, including all search terms and their combinations, is available in the online Supplementary Materials. All retrieved records were independently screened by two reviewers based on titles and abstracts to assess relevance and compliance with the reporting requirements. Studies were excluded if they met any of the following criteria: multiple publications derived from the same patient cohort or center (in which case only the most comprehensive report was retained); reviews, non-English publications; absence of molecular classification by NGS plus IHC methodology; or lack of available molecular data. For each included study, two investigators extracted data related to the EC case number and the corresponding sample size assigned to multiple-classifier categories: p53abn/TP53mut-MMRd/MSI, POLEmut-MMRd/MSI, POLEmut-p53abn/TP53mut, and POLEmut-MMRd/MSI-p53abn/TP53mut. Any discrepancies between investigators were resolved by consensus, with adjudication by a third reviewer when necessary. Study quality was evaluated according to the criteria of the Newcastle–Ottawa Scale.

### 2.4. Statistical Analysis

Meta-analysis was performed in R (version 4.x) using the meta package. All analyses employed a random-effects logistic regression model with logit transformation, which stabilizes variance when dealing with proportions close to 0 or 1. Between-study variance (τ^2^) was estimated using the maximum-likelihood method. For each study, proportions were calculated together with exact Clopper–Pearson confidence intervals. Both common-effect (fixed-effect) and random-effects models were computed. Between-study heterogeneity was quantified using τ^2^ and the I^2^ statistic, while statistical significance of heterogeneity was assessed using Wald and likelihood-ratio (LRT) Q-tests. I^2^ values were interpreted according to conventional thresholds: low (<25%), moderate (25–75%), and high (>75%). Comparisons between molecular groups were performed using Fisher’s exact test due to small sample size. Multi-categorical variables were dichotomized as follows: FIGO stage (III–IV vs. I–II), tumor grade (G3 vs. G1–G2), and LVSI (substantial vs. absent/focal).

## 3. Results

### 3.1. Clinical and Molecular Features of a Multiple-Classifier EC Cohort

Through the NGS and IHC combined methodological approach, the molecular classification of our EC series (n = 150) was performed, according to TCGA/ProMisE criteria (Table 1). Tumors were allocated into the following subgroups: POLEmut (n = 11, 7.4%), MMRd/MSI (n = 30, 20%), p53abn/TP53mut (n = 21, 14%), and NSMP (n = 79, 52.6%, this subgroup was not analyzed). As reported in our previous study [18], the assessment of MMRd and p53 status demonstrated high concordance between the IHC and NGS analyses, with agreement rates of 96.6% and 90.5%, respectively. One case showed weak and focal MSH6 staining by IHC, whereas NGS revealed extensive microsatellite instability. All p53-abnormal cases by IHC harbored pathogenic TP53 alterations by NGS, while two cases with TP53 frameshift mutations showed wild-type staining patterns. Overall, these results support IHC as a reliable screening tool for molecular classification and highlight the added value of NGS in resolving complex cases.

The clinicopathological characteristics of EC patients stratified by molecular subtypes are shown in Table 1. Multiple-classifiers were identified in 9 cases (6%). The predominant overlap was MMRd/MSI-p53abn/TP53mut (3.3%), followed by POLEmut-MMRd/MSI (2%). No POLEmut-p53abn/TP53mut case was observed, and a single case (0.7%) demonstrated triple positivity (combined presence of all three markers). As shown in Table 2, the POLEmut-MMRd/MSI group included three patients (EC1–EC3), ages 45–68 years, all with endometrioid histology and estrogen receptor (ER)-positive. The POLE mutations identified were D275G and V411L, associated with an ultramutated phenotype. The coexistence of multiple mechanisms of genomic instability were observed in patients EC1 and EC2, which exhibited defects in the MMR pathway and MSI associated with Lynch syndrome. Furthermore, the Comprehensive Genomic Profiling assay to determine TMB values showed that in all POLEmut-MMRd/MSI cases, TMB was markedly elevated (medium 411 muts/Mb), notably higher than in the MMRd/MSI-p53abn/TP53mut group. Clinically, following international guidelines, patients EC1 and EC3, with early-stage POLE-mutated disease, did not receive adjuvant treatment. Patient EC2 had grade 3, stage IIIa, cellular differentiation, and LVSI positivity. She did not receive any adjuvant treatment due to comorbidities and post-operative complications. Despite these risk factors, she is currently undergoing follow-up with a disease-free interval (DFI) of 25 months. Notably, in addition to the POLE gene mutation, the patient has a very high TMB (338.9/Mb). This real-world data confirmed that the POLE mutation and high TMB are associated with a better prognosis, even in the presence of other unfavorable risk factors.

The subgroup consisting of MMRd/MSI-p53abn/TP53mut tumors (EC5–EC9) displayed marked clinicopathologic heterogeneity. This group included both endometrioid and serous histotypes and was frequently associated with high-grade tumors, advanced FIGO stages, and LVSI positivity (three out of five cases), resulting in assignment to high-risk or advanced ESGO risk groups in most cases. Compared with POLEmut-MMRd/MSI tumors, TMB values in MMRd/MSI-p53abn/TP53mut tumors were more variable and generally lower (range 7–45 muts/Mb). Accordingly, adjuvant treatment in this subgroup most often consisted of combined chemotherapy and radiotherapy. EC6 and EC7 (FIGO stages IIIa and IIIc2) received adjuvant treatment according to the PORTEC-3 regimen and are currently disease-free after 17 months of follow-up. EC5 and EC9 were diagnosed EC at FIGO stage IV and treated with first-line chemo-immunotherapy (carboplatin, paclitaxel, and dostarlimab). However, EC5 died 4 months after treatment initiation for disease progression, EC9 showed an initial response, received subsequent lines of therapy, and died 27 months after diagnosis. During follow-up, EC9 also developed HR-positive breast cancer (BC), treated surgically and with adjuvant endocrine therapy. EC8 was diagnosed with synchronous endometrial and breast cancers at age 90; BC was treated with adjuvant endocrine therapy, while EC (FIGO stage II) was managed with adjuvant radiotherapy alone. She had a high TMB and is currently disease-free after 17 months of follow-up. The occurrence of metachronous malignancies in these patients suggested additional molecular complexity and a broader oncologic predisposition in this subgroup.

The triple-classifier EC (POLEmut-MMRd/MSI-p53abn/TP53mut, EC4) was observed in a single 82-year-old patient. The tumor was characterized by dedifferentiated histology and was classified as FIGO stage IIC, Grade 3. The patient also had a prior history of cutaneous squamous cell carcinoma. Despite the unfavorable histology and high grade, the case showed high TMB (90 muts/Mb) and was classified as low clinical risk according to the ESGO/ESTRO/ESP guidelines.

The correlation of molecular data to clinical outcomes was not performed because EC patients were predominantly in early disease stages and had a short follow-up period (median of entire cohort was 14 months).

### 3.2. Comparison Between Single- and Multiple-Classifiers EC

Comparison between single- and multiple-classifiers tumors are summarized in Table 1. The MMRd/MSI-p53abn/TP53mut group showed a significantly higher proportion of non-endometrioid histology compared with single-classifier MMRd/MSI tumors (*p* = 0.006). No significant difference in histotype distribution was observed between POLEmut-MMRd/MSI and either POLEmut or MMRd/MSI tumors. Tumor grade differed markedly across molecular subtypes. High-grade tumors (G3) were rare in POLEmut (27%) and MMRd/MSI tumors (23%) but predominated in p53abn/TP53mut tumors (95%). Similarly, the MMRd/MSI–p53abn/TP53mut group consisted exclusively of high-grade tumors, showing a significant difference compared with MMRd/MSI tumors (*p* = 0.001). Additionally, tumor grade differed significantly between POLEmut-MMRd/MSI and single-classifier MMRd/MSI tumors (*p* = 0.05). Advanced FIGO stage (III–IV) was uncommon in POLEmut (9%) and MMRd/MSI tumors (3%) but frequent in p53abn/TP53mut tumors (67%). The MMRd/MSI-p53abn/TP53mut group demonstrated a significantly higher proportion of advanced-stage disease compared with MMRd/MSI tumors (80% vs. 3%, *p* < 0.001), and POLEmut-MMRd/MSI tumors also showed significant differences relative to single-classifier MMRd/MSI tumors (*p* = 0.03). Lymph node metastases and substantial LVSI were significantly more frequent in p53abn/TP53mut tumors and in the MMRd/MSI-p53abn/TP53mut group compared with MMRd/MSI tumors (both *p* = 0.006 and *p* = 0.001, respectively). In contrast, POLEmut-MMRd/MSI tumors did not significantly differ from single-classifier POLEmut or MMRd/MSI tumors with respect to lymph node status or LVSI.

### 3.3. Literature Review

The literature review initially identified 164 records, of which 126—screened by title and abstract—were non-eligible. Following this screening, 38 articles underwent full-text evaluation. Of these, 11 studies met all inclusion criteria and were therefore included in the final analysis [10,12,15,20,21,22,23,24,25,26,27]. The study flowchart is reported in the Supplementary Materials (Table S1, Figure S1). All records were published between 2020 and 2025 (Table 3), seven were single-center, and four multi-centers. Most studies included all histologic types of EC, with one study restricted to high-risk tumors [24].

### 3.4. Multiple-Classifier Incidence

**Figure 1:**
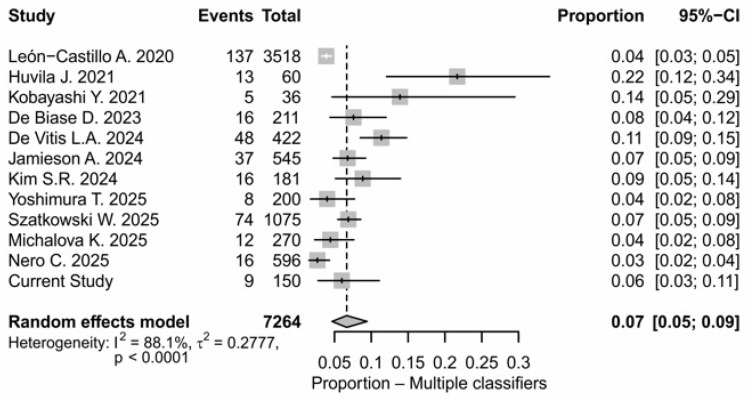
Forest plot of the proportion of multiple-classifier ECs across the included studies and the current cohort. The *x*-axis displays proportions (0–1 scale). The diamond indicates the random-effects pooled estimate, with heterogeneity reported as I^2^, τ^2^, and *p*-values.

**Figure 2:**
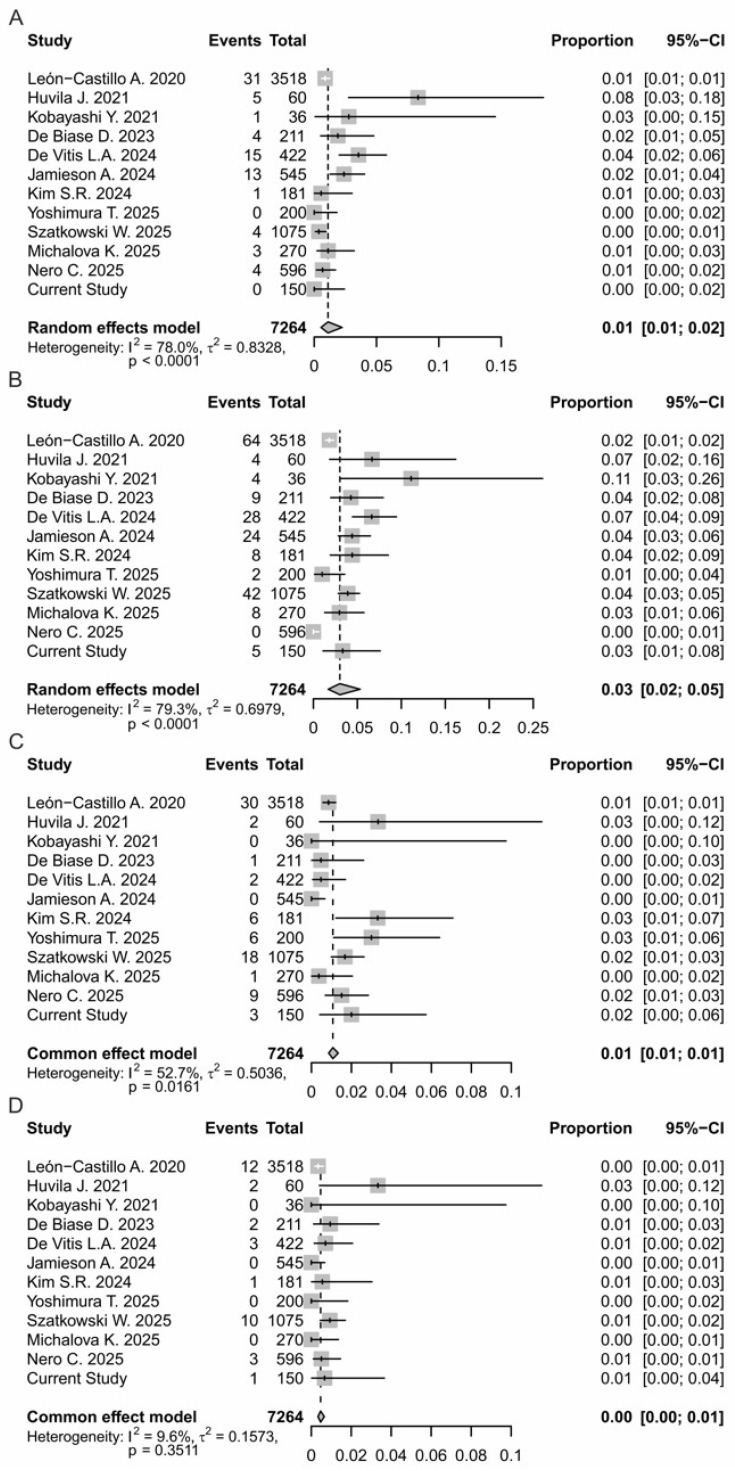
Forest plots showing the pooled prevalence of multiple-classifier ECs across the included studies and the current cohort. (**A**) POLEmut–p53abn/TP53mut ECs; (**B**) MMRd/MSI-p53abn/TP53mut ECs; (**C**) POLEmut-MMRd/MSI ECs; (**D**) Triple-classifier ECs (POLEmut-MMRd/MSI-p53abn/TP53mut). For each study, the number of events and total cases, estimated proportions with 95% confidence intervals (CIs), and study weights are reported. Diamonds represent pooled prevalence estimates calculated using random-effects models in the presence of high heterogeneity and fixed-effects models when heterogeneity was moderate or low. Between-study heterogeneity is expressed as I^2^, τ^2^, and *p* values.

To evaluate the incidence of multiple-classifier ECs, a meta-analysis of 12 studies including a total of 7264 patients was performed (Table 3). The percentage of multiple classifiers across all patients was 5.4%. As shown in the forest plot in Figure 1, the pooled estimated proportion of the event across all included studies was 0.05 (95% CI: 0.05; 0.06). Individual study prevalence varied widely, from a minimum of 2.7% [22] to a maximum of 21.5% [26], and between-study heterogeneity was high (I^2^ = 88.1%), suggesting substantial differences in assessment criteria or study populations (Figure 1 and Figure 2). Meta-analytic models were selected based on the degree of heterogeneity: random-effects models were applied when I^2^ was high (>75%) to account for variability among studies, while fixed-effects models were used for subgroups with moderate or low heterogeneity. In particular, the combinations of MMRd/MSI-p53abn/TP53mut and POLEmut-p53abn/TP53mut had an estimated proportion of 0.03 (95% CI: 0.02; 0.05), ranging from 0% [22] to 11.1% [24] and 0.01 (95% CI: 0.01; 0.02) and from 0% [27] to 8.3% [26], respectively. Heterogeneity was high (I^2^ = 79.4% and 78.0%), so meta-analysis was performed using a random-effects model (Figure 2B). The POLEmut-MMRd/MSI combination was rare, with a pooled prevalence of 0.01 (95% CI: 0.01; 0.02), and moderate heterogeneity (I^2^ = 52.7%), so meta-analysis was performed using a fixed-effects model (Figure 2C). This indicated more consistency between studies despite low event frequency. In our cohort, no cases with the molecular classification POLEmut-p53abn/TP53mut were identified (Figure 2A). In the literature, the frequency of this subgroup ranges from 0% to 8.3%, with the highest incidence reported by Huvila et al. [26]. Other studies, such as León-Castillo et al. [10] and De Biase et al. [23], reported lower or similar frequencies around 4%. Regarding the triple positivity (POLEmut-MMRd/MSI-p53/TP53mut) ECs, these were exceptionally rare and observed in less than 0.5% of patients. Heterogeneity was minimal (I^2^ = 9.6%), suggesting that the prevalence of this rare alteration is consistent across studies (Figure 2D).

Although formal subgroup or sensitivity analyses were limited by the small number of events for some molecular combinations, separate analyses for each classifier combination were performed to explore potential differences and assess the robustness of the findings. In addition, a leave-one-out sensitivity analysis was conducted for the overall multiple-classifier EC group. Omitting each study in turn did not substantially change the pooled proportion (remaining around 5–6%), indicating that no single study disproportionately influenced the meta-analytic estimate. Despite high heterogeneity (I^2^ ≈ 88%), the results were consistent across studies, supporting the robustness of the findings.

## 4. Discussion

EC classification has progressively evolved through the integration of conventional histopathology with molecular profiling, resulting in improved risk stratification and more individualized therapeutic approaches [3,4,5,6,7,8,9]. Within the framework of precision oncology, the identification of molecular subtypes and predictive biomarkers has become essential for treatment selection and prognostic assessment [28,29]. Despite these advances, the identification of tumors harboring more than one molecular classifier has revealed important limitations of current classification systems, particularly with respect to prognostic stratification and therapeutic decision-making. Although current algorithms apply hierarchical rules to simplify classification, growing evidence suggests that tumors harboring multiple-classifiers represent biologically heterogeneous entities rather than a uniform group [12]. The present study contributes to this evolving field by providing clinicopathological characterization of multiple-classifier ECs and by estimating their incidence through a systematic review and meta-analysis that incorporates our cohort data.

Primarily, we expanded our EC dataset by integrating molecular and clinicopathological data previously published [18] in an additional independent EC patient cohort. Overall, 150 EC patients were categorized in the four molecular subtypes by NGS and IHC, with the following distribution: POLEmut 7.4%, MMRd/MSI 20%, p53abn/TP53mut 14%, and NSMP 52.6% (Table 1). Additionally, 6% of all EC cases fell into the multiple-classifier category harboring more than one molecular classifying feature and including POLEmut-MMRd/MSI, MMRd/MSI-p53abn/TP53mut, and POLEmut-MMRd/MSI-p53abn/TP53mut (Table 1 and Table 2). Subsequently, we compared their clinicopathological features with those of single classifiers sharing one molecular feature. Our findings showed that two cases out of three classified as POLEmut-MMRd/MSI displayed hallmark features of POLE-driven tumors including younger patient age (mean age of 55 years), predominance of endometrioid histology, low-grade morphology, early-stage presentation, and limited LSVI. The third case was characterized by more aggressive features at diagnosis (Grade 3, LSVI positive) and did not receive adjuvant therapy because of comorbidities; nevertheless, the patient remains disease-free at 25 months of follow-up. Notably, the POLE mutations identified (D275G and V411L) were well-established hotspot variants associated with an ultramutated phenotype. The coexistence of multiple mechanisms of genomic instability was observed in EC1 and EC2, which exhibited defects in the MMR pathway and MSI associated with Lynch syndrome [30,31] (Table 2). The Comprehensive Genomic Profiling assay further demonstrated that all POLEmut–MMRd/MSI cases showed markedly elevated TMB values (>300 muts/Mb, Table 2), notably higher than those observed in the MMRd/MSI–p53abn/TP53mut group. Previous clinical data have shown that in tumors with multiple molecular classifiers, the classifier conferring the highest TMB—typically POLE or dMMR—dominates the immunogenic phenotype and predicts favorable immune checkpoint inhibitor (ICI) response [32]. Consistent with several studies [10,33,34,35,36], our observations suggest that these dual-classifier tumors generally retain a typically POLEmut ultramutated profile, which is associated with strong immunogenicity and favorable prognosis.

Differently, ECs harboring concurrent MMRd/MSI and p53/TP53 abnormalities exhibited an aggressive pathological profile. Although León-Castillo et al.’s study [10] reported favorable recurrence-free survival in MMRd-p53abn tumors, more recently, Szatkowski et al. [20] showed a more aggressive profile compared with MMRd-only tumors, with higher rates of non-endometrioid histology, grade 3 disease, and high–intermediate risk according to ESGO/ESTRO/ESP. Similarly in the work by Michalova et al. [15], three-fifths of patients developed metastatic disease, and one died. De Vitis et al.’s study [12] also observed a trend toward increased recurrence in MMRd-p53abn tumors, although this did not reach statistical significance. In line with these observations, in our cohort, MMRd/MSI-p53abn/TP53mut tumors exhibited uniformly high-grade morphology, advanced FIGO stage, lymph node involvement, and positive LVSI. In comparison with single-classifier MMRd/MSI tumors, this subgroup exhibited adverse clinicopathologic features, suggesting that p53/TP53 alterations might worsen the typical intermediate-risk behavior of MMRd ECs. This finding supports emerging evidence that co-occurring p53/TP53 mutations define a biologically aggressive phenotype, even in tumors otherwise classified as MMRd/MSI. Compared with POLEmut-MMRd/MSI (Table 2), MMRd/MSI-p53abn/TP53mut tumors exhibited lower and more heterogeneous TMB values—from intermediate to low values—suggesting their less favorable prognosis and the need for intensified adjuvant treatment. In TMB-low subtypes, the response to ICIs may be limited, and combined strategies are required to achieve clinical benefit [37]. Indeed, in our cohort, MMRd/MSI-p53abn/TP53mut patients with localized disease (EC6–EC8) received combined chemotherapy and radiotherapy, reflecting current clinical practice for high-risk diseases. The two patients with metastatic disease (EC5, EC9) were treated with chemotherapy and immunotherapy. Nevertheless, clinical outcomes remained variable; the patients with localized disease did not experience disease recurrence, while the metastatic patients both died. Notably, EC5 showed a rapidly progressive course, with progression within three months and death shortly, thereafter, highlighting that the coexistence of MMRd/MSI and p53/TP53 alterations may confer more aggressive behavior than typically expected for MSI-induced tumors alone. The development of metachronous primary BC in two patients (EC8 and EC9) further underlined oncologic predisposition and increased molecular complexity within this subgroup. The association between EC and BC is likely multifactorial, reflecting shared hormonal risk factors—such as estrogen exposure related to nulliparity, early menarche, and late menopause—as well as obesity and underlying genetic susceptibility [38,39]. Regarding triple-classifier POLEmut-MMRd/MSI-p53abn/TP53mut ECs, they were exceedingly rare in both the literature and our cohort. The limited number of reported cases precludes definitive conclusions regarding prognosis or optimal management. Notably, the patient harboring this molecular profile had a prior primary malignancy, specifically cutaneous squamous cell carcinoma. Although no link between these tumors has been reported, this occurrence may reflect, at least in part, an underlying genetic susceptibility related to complex molecular alterations such as p53/TP53 abnormalities.

Furthermore, to investigate the incidence of multiple-classifier ECs, we combined evidence from a systematic review and meta-analysis that included our cohort (Figure 1 and Figure 2). This analysis demonstrated that multiple-classifier tumors represent a small but consistent proportion of ECs, with an overall prevalence of approximately 5.4% (Figure 1). Among the observed molecular overlaps, MMRd/MSI-p53abn/TP53mut tumors were the most frequently reported (Figure 2), although heterogeneous, suggesting different biological pathway activations or tumor evolution stages. In contrast, POLEmut- p53abn/TP53mut tumors appeared less common and more variably reported (Figure 2), likely reflecting differences in the p53/TP53 interpretation criteria. In line with some published series, in our cohort, POLEmut-p53abn/TP53mut tumors were absent (Figure 2). The POLEmut-MMRd/MSI combination emerged as an uncommon but more consistently reported entity, as reflected by moderate heterogeneity (Figure 2). Triple-classifier ECs (POLEmut-MMRd/MSI-p53abn/TP53mut) were exceedingly rare, occurring in less than 0.5% of patients, with minimal heterogeneity across studies (Figure 2). Although their low frequency limits definitive conclusions regarding prognosis and optimal management, their existence raises important questions about molecular hierarchy and the relative prognostic weight of competing genomic alterations, particularly in the context of risk stratification and treatment decision-making. Our findings are consistent with the recent meta-analysis by De Vitis et al. [12], who reported an overall prevalence of multiple-classifier ECs ranging from 1.8% to 9.8% among studies including more than 100 patients. Similarly, in our analysis of larger cohorts, the incidence ranged from 2.7% to 11.4%. The slight differences in upper incidence limits are likely attributable to the use of combined NGS and IHC in selected series, which likely improves the detection of concurrent molecular alterations. Importantly, our results reinforce and extend the observations of De Vitis et al. by providing a statistically robust confirmation that inter-study variability in the reported multiple-classifier EC prevalence is driven mainly by methodological heterogeneity. Notably, by restricting our analysis to studies adopting relatively uniform molecular testing strategies, substantial heterogeneity persisted. This variability likely reflects differences in NGS panel size and gene coverage, analytical sensitivity, interpretative criteria applied to POLE and TP53 variants as well as IHC use versus sequencing-based approaches [40,41,42,43]. In line with the literature that reports a broader range (60–92.3%) for p53abn/TP53mut detection, in our cohort, the concordance between NGS and IHC was 90.5%. This discrepancy was likely attributable to suboptimal tissue processing and subclonal or atypical p53 IHC staining, which are difficult to detect by only IHC [44,45]. Overall, our observations highlight the need for harmonized molecular testing strategies and standardized reporting criteria to ensure consistent classification of multiple-classifier ECs.

Indeed, this study has several limitations. Multiple-classifier ECs, particularly triple-classifier tumors, are extremely rare, limiting robust conclusions on prognosis and management. Besides the methodological heterogeneity, the inclusion of unselected patient populations—with heterogeneous histology, stage, and risk—complicate direct comparisons across studies. Additionally, follow-up was relatively short in some cases, which may have underestimated late events and precluded meaningful survival analyses. Moreover, TMB was assessed only in a small subset of cases (n = 9) using a dedicated sequencing platform; therefore, any observations regarding TMB-related biology should be considered exploratory and interpreted with caution. These limitations highlight the need for large, well-annotated cohorts and standardized molecular testing to clarify the clinical significance of multiple-classifier ECs. Nonetheless, this work provides a large, systematically analyzed dataset, integrates our cohort findings with a comprehensive meta-analysis, and offers statistically robust, data-driven insights into the prevalence and complexity of multiple-classifier ECs, supporting improved molecular classification and future risk stratification.

## 5. Conclusions

This study demonstrates that multiple-classifiers represent a small but consistent subset of ECs, accounting for approximately 5% of cases, and encompass biologically heterogeneous entities rather than a uniform group. In our EC cohort, POLEmut tumors with concurrent MMRd/MSI generally retained the POLE-associated ultramutated profile. Tumors with both MMRd/MSI and p53abn/TP53mut alterations were more often associated with more adverse clinicopathological characteristics compared to MMRd/MSI-only tumors, highlighting the limitations of current hierarchical molecular classifications. By integrating our cohort with a systematic review and meta-analysis, we show that the variability in the reported incidence of multiple-classifier ECs is procedure-driven, underscoring the importance of standardized molecular testing and interpretation criteria. Overall, these findings highlight the need for harmonized diagnostic strategies to ensure the accurate classification and optimal clinical management of multiple-classifier ECs.

## Figures and Tables

**Table 1:** Clinicopathological characteristics of EC patients (n = 150) according to molecular profile and comparison between single and multiple-classifier tumors. Triple positive subgroup included only a single case, so statistical analysis could not be performed and is therefore not shown in the table.

	POLEmut n = 11	MMRd/ MSI n = 30	p53abn/TP53mut n = 21	POLEmut- MMRd/MSI n = 3	MMRd/MSI -p53abn/TP53mut n = 5	POLEmut-MMRd/MSI vs. POLEmut *p*-Value	POLEmut- MMRd/MSI vs. MMRd/MSI *p*-Value	MMRd/MSI– p53abn/TP53mut vs. MMRd/MSI *p*-Value	MMRd/MSI–p53/TP53mut vs. p53abn/TP53mut *p*-Value
**Age, years**	55 ± 10	65 ± 11	70 ± 9	55 ± 11	62 ± 16				
**Histotype**						0.58	0.74	0.006	0.06
Endometrioid	10 (91%)	29 (97%)	4 (19%)	3 (100%)	3 (60%)				
No endometrioid	1 (9%)	1 (3%)	17 (81%)	0	2 (40%)				
**Grade**						0.83	0.05	0.001	0.61
G1–G2	8 (73%)	23 (77%)	1 (5%)	2 (66%)	0				
G3	3 (27%)	7 (23%)	20 (95%)	1(33%)	5 (100%)				
**FIGO stage**						0.28	0.03	<0.001	0.56
I–II	10 (91%)	29 (97%)	7 (33%)	2 (66%)	1 (20%)				
III–IV	1 (9%)	1 (3%)	14 (67%)	1(33%)	4 (80%)				
**Lymph node status**						0.58	0.74	0.006	0.31
Negative	10 (91%)	30 (100%)	17 (81%)	3 (100%)	3 (60%)				
Positive	1 (9%)	0	4 (19%)	0	2 (40%)				
**LVSI**						0.28	0.36	0.001	0.56
Absent/Focal	10 (91%)	26 (87%)	6 (29%)	2 (66%)	1 (20%)				
Substantial	1 (9%)	4 (13%)	15 (71%)	1(33%)	4 (80%)				

**Table 2:** Clinicopathological and molecular features of multiple-classifier EC patients. * Patient died.

Patients	EC1	EC2	EC3	EC4	EC5 *	EC6	EC7	EC8	EC9 *
**Age at time of** **diagnosis**	45	54	68	82	56	61	55	90	51
**Histotype**	endometrioid	endometrioid	endometrioid	dedifferentiated	endometrioid	endometrioid	endometrioid	serous	endometrioid
**Multiple-** **classifier EC**	POLEmut-MMRd/ MSI	POLEmut-MMRd/ MSI	POLEmut-MMRd/ MSI	POLEmut- MMRd/MSI -p53abn/ TP53mut	MMRd/MSI- p53abn/ TP53mut	MMRd/MSI- p53abn/TP53mut	MMRd/MSI- p53abn/TP53mut	MMRd/MSI- p53abn/TP53mut	MMRd/MSI- p53abn/TP53mut
**POLE variant**	V411L	D275G	D275G	V411L	WT	WT	WT	WT	WT
**FIGO stage, GRADE**	Ia3, G2	IIIA, G3	IA, G2	IIC, G3	IVb, G3	IIIa, G3	IIIC2, G3	II, G3	IVb, G3
**N**	No	no	no	no	yes	no	yes	no	no
**LVSI**	No	yes	no	no	yes	no	yes	yes	yes
**ER**	Yes	yes	yes	no	no	yes	yes	no	yes
**TMB (Muts/Mb)**	410	339	485	90	7	15	20	45	7
**ESGO** **Clinical Risk Group**	low risk	uncertain	low risk	low risk	advanced	high risk	high risk	high risk	advanced
**Hereditary disease**	Lynch syndrome	Lynch syndrome	no	no	no	no	no	no	no
**Relapse**	No	yes	no	no	yes	no	no	no	no
**Other neoplasm**	No	no	no	Squamous cell carcinoma of the skin	no	no	no	BC (lobular carcinoma, pT1c pN0, G2, HR+, HER2 2+ (non-amplified FISH), Ki67 5%)	BC (infiltrating ductal carcinoma pT1c pN0, G2, HR-positive, HER2-negative, Ki67 15%)
**DFI months**	16 +	25	2 +	6+	/	17+	17+	27+	/
**Treatment**	No	no	no	no	Carboplatin, paclitaxel and dostarlimab (4 mo)	Adjuvant: cisplatin for two cycles + RT + 4 cycles of carboplatin and paclitaxel	Adjuvant: cisplatin for two cycles + RT + 4 cycles of carboplatin and paclitaxel	RT	Carboplatin, paclitaxel and dostarlimab (12 mo) + doxorubicin (5 mo) + paclitaxel (6 mo)

**Table 3:** Studies on multiple-classifier ECs assessed by the NGS and IHC methods.

Studies	Multi- Institutional	N. Patients	Multiple- Classifiers	p53abn/TP53mut-MMRd/MSI	POLEmut-p53abn/TP53mut	POLEmut-MMRd/ MMRd	Triple Positive
León-Castillo, 2020 [10]	yes	3518	3.9% (137/3518)	1.8% (64/3518)	0.9% (31/3518)	0.9% (30/3518)	0.3% (12/3518)
Huvila, 2021 [26]	no	60	21.6% (13/60)	6.6% (4/60)	8.3% (5/60)	3.3% (2/60)	3.3% (2/60)
Kobayashi, 2021 [24]	no	36	13.90% (5/36)	11.1% (4/36)	2.8%(1/36)	0%	0%
De Biase, 2023 [23]	no	211	7.60% (16/211)	6.6% (9/211)	4.2% (4/211)	0.47% (1/211)	0.94% (2/211)
De Vitis, 2024 [12]	no	422	11.40% (48/422)	6.6% (28/422)	3.6% (15/422)	0.5% (2/422)	0.7% (3/422)
Jamieson, 2024 [25]	yes	545	6.8% (37/545)	4.4% (24/545)	0.8% (13/545)	0%	0%
Kim, 2024 [21]	yes	181	8.80% (16/181)	4.4% (8/181)	0.5% (1/181)	3.3% (6/181)	0.5% (1/181)
Yoshimura, 2025 [27]	no	200	4% (8/200)	1% (2/200)	0%	3% (6/200)	0%
Szatkowski, 2025 [20]	no	1075	6.90% (74/1075)	3.9% (42/1075)	0.4% (4/1075)	1.7% (18/1075)	0.9% (10/1075)
Michalova, 2025 [15]	no	270	4.40% (12/270)	3% (8/270)	1.1% (3/270)	0.3% (1/270)	0%
Nero, 2025 [22]	no	596	2.7% (16/596)	0%	3% (4/596)	1.1% (9/596)	0.3% (3/596)
Current Study	no	150	6% (8/150)	3.33% (5/150)	0%	2% (3/150)	0.6% (1/150)

Information regarding the methodology to assess p53/TP53 status (IHC and/or TP53 sequencing) was not consistently provided in the included studies.

## Data Availability

The data presented in this study are available upon request from the authors.

## Supplementary Materials

The following supporting information can be downloaded at: https://www.mdpi.com/article/10.3390/cancers18060929/s1, Table S1: Search strategy for PubMed database. Figure S1: PRISMA flow diagram showing the study selection process for the systematic review.
