# Supplementary material for: Prognostic Stratification of Multiple-Classifier Endometrial Cancers: Cohort Study and Meta-Analysis
Source: Cancers (Basel). 2026 Mar 12;18(6):929. doi: 10.3390/cancers18060929 (PMC13024139; doi:10.3390/cancers18060929)
Supplement: Supplementary file 1 [file cancers-18-00929-s001.zip › cancers-4179713-supplementary.pdf]

Table S1.**Search Strategy for PubMed Database**

ID	Search query	Filters	Query PubMed	Results
**3**	(“Endometrial Neoplasms”[Title/Abstract] OR “endometrial cancer”) AND (classifier)	Most Recent	(“Endometrial Neoplasms”[Title/Abstract] OR “endometrial cancer”[All Fields]) AND (“classifiable”[All Fields] OR “classification”[MeSH Terms] OR “classification”[All Fields] OR “classified”[All Fields] OR “classify”[All Fields] OR “classifying”[All Fields] OR “classifier”[All Fields] OR “classifier s”[All Fields] OR “classifiers”[All Fields] OR “classifies”[All Fields])	**2,066**
**4**	(“Endometrial Neoplasms”[Title/Abstract] OR “endometrial cancer”) AND (classifier)	2016–2026	(…query 3…) AND (2016:2026[pdat])	**1,493**
**5**	(“Endometrial Neoplasms”[Title/Abstract] OR “endometrial cancer”) AND (multiple classifier)	Most Recent	(“Endometrial Neoplasms”[Title/Abstract] OR “endometrial cancer”[All Fields]) AND ((“multiple”[All Fields] OR “multiples”[All Fields]) AND (classific* terms…))	**157**
**8**	(“Endometrial Neoplasms”[Title/Abstract] OR “endometrial cancer”) AND (multiple classifier)	2016–2026	(…query 5…) AND (2016:2026[pdat])	**131**
**15**	(“Endometrial carcinoma”[Title/Abstract] OR “endometrial cancer”[Title/Abstract]) AND “multiple classifier”[Title/Abstract]	Most Recent	(…stringa identica…)	**14**
**18**	(“Endometrial carcinoma” OR “endometrial cancer”) AND (“multiple-classifier” OR “concomitant”)	Most Recent	(“Endometrial carcinoma”[Title/Abstract] OR “endometrial cancer”[Title/Abstract]) AND (“multiple-classifier”[All Fields] OR “concomitant”[All Fields])	**315**
**19**	Come query 18	2015–	(…query 18…) AND (2015:2025[pdat])	**144**

ID	Search query	Filters	Query PubMed	Results
		2025		
**17**	(“Endometrial carcinoma” OR “endometrial cancer”) AND “multiple-classifier”	Most Recent	(“Endometrial carcinoma”[Title/Abstract] OR “endometrial cancer”[Title/Abstract]) AND “multiple-classifier”[All Fields]	**14**
**20**	(“Endometrial carcinoma” OR “endometrial cancer”) AND (“multiple-classifier” OR “POLEmut-MRRd” OR “MMRd-p53abn”)	Most Recent	(“Endometrial carcinoma”[Title/Abstract] OR “endometrial cancer”[Title/Abstract]) AND (“multiple-classifier”[All Fields] OR (“POLEmut”[All Fields] AND “MRRd”[All Fields]) OR “MMRd-p53abn”[All Fields])	**23**
**21**	Come query 20 + “POLEmut-p53abn”	Most Recent	(…query 20…) OR “POLEmut-p53abn”[All Fields]	**24**

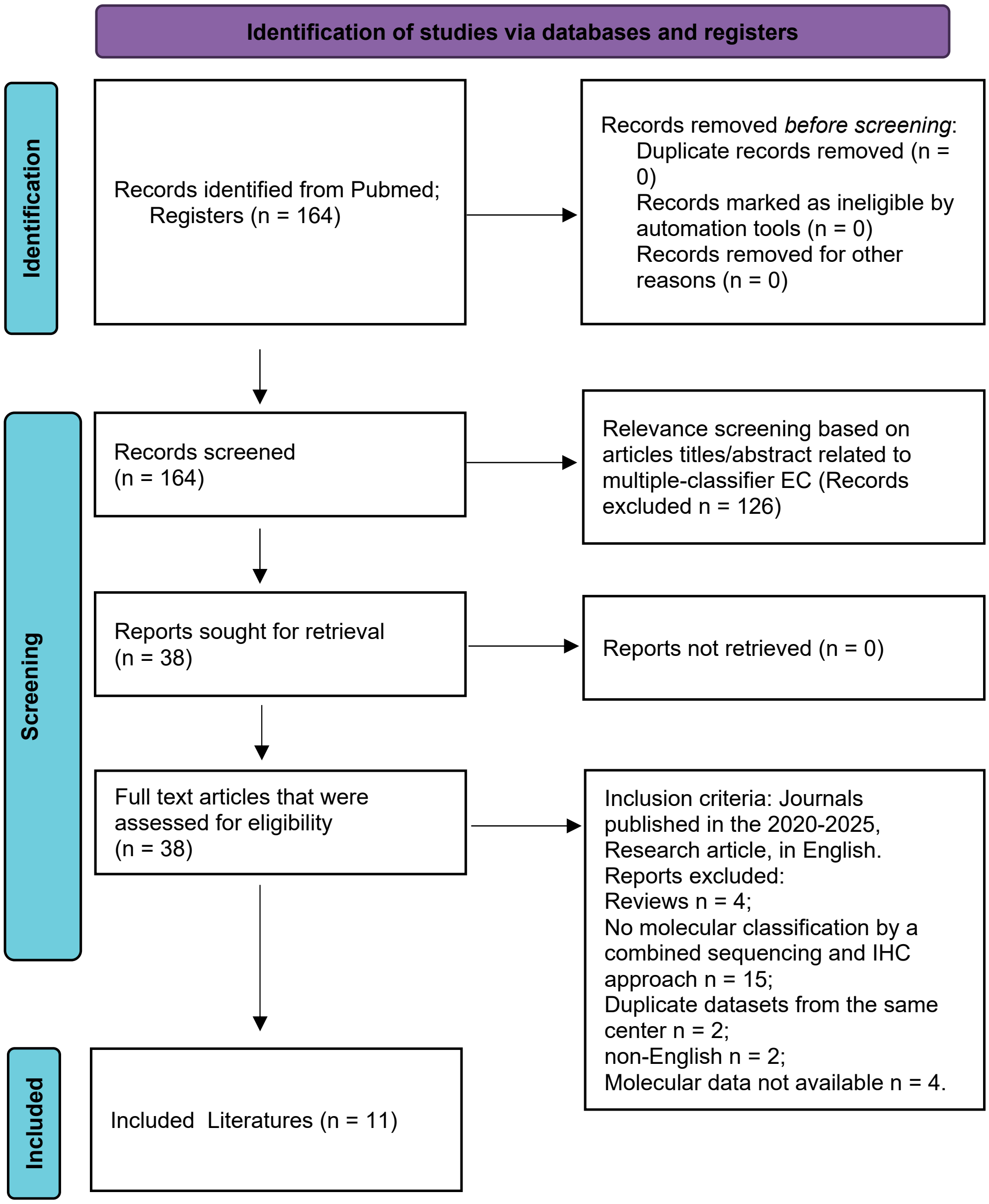

Figure S1. PRISMA flow diagram showing the study selection process for the systematic review.
